# A Singular Case

**Published:** 1883-08

**Authors:** 


### A Singular Case.

In a private letter recently from Prof. Watling, of Ypsilanti, Michigan, is described a novel affection. Of it, he says: "It has proved to be a very interesting case. I have had it on hand for some time, and I am likely to, I fear for some time to come. It is in the mouth of a lady about forty-five years of age, and presents an opening through the gum of the lower jaw, at about the point where the third molar should be. The opening is from one-third to one-half an inch in diameter, and the passage within nearly as large. The canal passes backward and upward, just on the outside of the ramus of the jaw, and back into the cheek, to a point nearly opposite the middle of the ear, where there is a large cavity in the location of the parotid gland. It has the appearance of the breaking down and wasting away of this gland. When I began treating it quite large pieces of yellowish material, resembling gland substance came away, but so soft and broken down that the microscope revealed little or nothing of structural character. The opening and canal has existed for two or three years, the general health is not disturbed by it, there is not much pain at present, nor has there ever been severe pain. There is no appreciable discharge from the opening. I am greatly interested to know just the nature of the case. What is the matter? Is it possible for the parotid gland to break down and waste away in that manner? The duct of steno is closed. I have consulted with some of our physicians and they are as much in the dark as to the cause of the trouble and its nature as I am."

I recently saw this case, and found it about as described above. The health of the patient is good, and the part affected is evidently improving by the manner of treatment employed, which is occasionally washing with mild disinfectant and antiseptic preparations, no general treatment has seemed to be required.

Is it possible that the parotid gland could be thus destroyed without producing more disturbance than has existed in this case?

